# Selective cerebral perfusion with 4-branch graft total aortic arch replacement: Outcomes in 12 patients

**DOI:** 10.1186/1749-8090-7-32

**Published:** 2012-04-13

**Authors:** Wei-Liang Lai, Chiao-Po Hsu, Chung-Che Shih, Ming-Li Li, Ping-chun Li

**Affiliations:** 1 Division of Cardiovascular Surgery, Department of Surgery, Taipei Veterans General Hospital, No. 201, Sec. 2, Shih-Pai Rd., Taipei 112, Taiwan, Republic of China; 2 Division of Cardiovascular Surgery, Department of Surgery, China Medical University Hospital, Taipei, Taiwan, Republic of China; 3 China Medical University, Taichung, Taiwan, Republic of China

**Keywords:** Aortic arch aneurysm, Type A aortic dissection, Branched aortic graft

## Abstract

**Background:**

Aortic arch reconstruction is associated with high neurological morbidity. Our purpose is to describe our experience using a 4-branched graft and selective antegrade brain perfusion (SABP) for total aortic arch replacement (TAR).

**Methods:**

We retrospectively reviewed the medical records of 12 patients who received TAR, with or without ascending aorta replacement, with a 4-branched graft for Stanford type A dissection (*n *= 9) or aortic arch aneurysm (*n *= 3). In all patients surgery was performed with deep hypothermic circulatory arrest (DHCA) with or without retrograde brain perfusion, and selective antegrade brain perfusion (SABP) via the subclavian artery or axillary artery.

**Results:**

There were 8 males and 4 females with an average age of 63.14 years. Emergent operations were performed in 9 patients with acute type A aortic dissections. Of all 12 patients, 2 deaths occurred and 1 patient experienced lower extremity paraplegia resulting in an in-hospital mortality rate of 16.6% and a permanent neurological deficit rate of 8.3%.

**Conclusions:**

The use of a 4-branched graft, hypothermic circulatory arrest, and SABP is a useful operative method for aortic arch replacement with acceptable morbidity and mortality.

## Background

Aortic arch surgery is associated with significant morbidity and mortality, and neurologic dysfunction remains a major complication of operations that involve total aortic arch replacement (TAR). There are 2 general methods of protecting the brain during extensive surgical procedures, deep hypothermic circulatory arrest (DHCA) with or without retrograde brain perfusion, and selective antegrade brain perfusion (SABP) via the subclavian artery or axillary artery [1,2]. DHCA should not exceed 45 to 50 min, but provides a dry operation field. SABP introduces the risk of embolization of atheromatous debris or air, but provides oxygenated blood to the brain and reduces pump time and the risk of hypothermia-related complications such as pulmonary insufficiency and coagulopathy [3-5]. Therefore, SABP is currently considered to be the most reliable method of preventing brain ischemia [6].

Many techniques have been developed for replacement of the aortic arch, and the choice often relies on the unique clinical situation of a patient and surgeon experience [7-10]. Among the many techniques, authors have reported TAR using a 4-branched prosthetic graft, SABP, and hypothermia is associated with good outcomes [11,12].

The purpose of this report is to describe our experience using a 4-branched graft, DHCA, and SABP for TAR.

## Methods

In this retrospective study, the medical records of 12 patients who underwent aortic arch reconstruction with 4-branched grafts, with or without ascending aorta replacement or Bentall procedure were reviewed. The patients were from 2 medical centers, Taipei Veterans General Hospital and China Medical University Hospital, and all received surgery during the period from February 2006 to January 2010. This study was approved by the Institutional Review Boards of the hospitals, and because of its retrospective nature the requirement of informed patient consent was waived.

### Surgical procedures

Dissection and cannulation of the right axillary artery was performed prior to median sternotomy in 9 patients. Median sternotomy was performed in 11 patients and bilateral thoracotomy in 1 patient. The arterial cannula was inserted via the right axillary artery. One 2-stage venous cannula was used for drainage of venous flow via the right auricle. After initiation of cardiopulmonary bypass the patient was cooled. Myocardial protection was based on antegrade perfusion of either crystalloid or blood cadioplegic solution, with or without continuous retrograde perfusion, via the coronary sinus. Circulation was arrested at a rectal temperature below 23°C, and the aortic arch was opened after accessing the branch vessels. Selective cerebral perfusion via the right axillary artery was maintained at 10 mL/min/kg or 1000 mL/min depending on the surgeon's preference. All arch reconstructions and distal anastomoses were performed with an open technique with low-flow perfusion through the right axillary artery.

Branched vessels of the aortic arch were identified and looped. First, the brachiocephalic trunk was clamped and cut down. Then, an anastomosis was made between the branched graft and the brachiocephalic trunk. At the same time, right-side cerebral perfusion was maintained from the right subclavian cannula, and left-side cerebral perfusion was maintained from the aorta to avoid cerebral ischemia. Next, the left common carotid artery was anastomosed with the branched graft while left-side cerebral perfusion was temporarily decreased due to clamping. After anastomoses were completed, bilateral cerebral perfusion was established from the arterial cannula at the right subclavian artery through the branched graft.

After bilateral cerebral perfusion has been established, the ascending aorta was crossclamped, and cardiac arrest with cardioplegia and cooling performed. The proximal descending aorta distally to the aneurysm was transected completely with care to not injury the phrenic and vagal nerves.

An open distal anastomosis was performed during DHCA with selective cerebral perfusion. Woven Dacron vascular grafts sealed with collagen (Hemashield; Meadox Medical, Oakland, NJ, USD) were directly anastomosed to the descending aorta with 3-0 polypropylene continuous suture with or without using the reverse sleeve method. Upon completion of the anastomosis, any debris or air in the descending aorta was evacuated. Then, the left subclavian artery was anastomosed with the graft. Cerebral perfusion via the 4^th ^branch of the graft was initiated after the completion of the left common carotid artery anastomosis. Rewarming was started during anastomosis of the brachiocephalic artery. Finally, the proximal end of the branched graft was anastomosed with the ascending aorta. Concomitant procedures included the Bentall operation in 3 patients. These patients were diagnosed as Stanford type A aortic dissections in which the coronary arteries and aortic valve were involved.

### Statistical analysis

Continuous data are shown as the mean (range), while categorical data are represented by frequency. The descriptive statistics were performed using SPSS 15.0 statistics software (SPSS Inc., Chicago, IL, USA).

## Results

### Patient and operative data

The mean age of the 12 patients was 63.1 years (range, 42-85 years), and there were 8 females and 4 males (Table 1). The mean follow-up period of all patients was 39.2 months (range, 0.5 to 6.3 years).

**Table 1:** Patient characteristics (N = 12)

Male/Female	8/4
Age (years)	63.1 (42-85)

Diagnosis	

Acute type A aortic dissection	9

Aortic arch aneurysm	3

Hypertension	10

Diabetes	1

Chronic obstructive pulmonary disease	2

Old cerebral vascular accident	3

Marfan syndrome	2

Chronic renal insufficiency	2

Data are presented a number or mean (range)

Emergent operations were performed in 9 patients with acute type A aortic dissections involving the ascending aorta to the aortic arch, and among these 9 patients the inlet of the dissection was over the lesser curvature of aortic arch in 6 patients and distal to the left subclavian artery in the other 3 patients (Table 2). Urgent operations were performed in 3 patients with aortic arch aneurysms. The 2 patients with Marfan syndrome received surgery due to extended aneurysm formation over the aortic arch area after a prior Bentall procedure.

**Table 2:** Surgical and cardiopulmonary perfusion data (N = 12)

Surgical procedure	
Ascending aorta replacement + TAR	12

Concomitant Bentall operation	3

Cardiopulmonary perfusion data	

Cardiopulmonary bypass time (min)	188.9 (158-247)

Aortic crossclamping time (min)	121.3 (96-162)

DHCA + SABP (min)	64.1 (48-82)

TAR, total aortic arch replacement; DHCA, deep hypothermic circulatory arrest; SABP, selective antegrade brain perfusion

Data are presented a number or mean (range)

Four patients received 26 mm grafts, 5 received 28 mm grafts, and 30 mm grafts were used in 3 patients. There was no obvious mismatch between the vessels and the branched graft in any patient. The mean total cardiopulmonary bypass time was 188.9 min (range, 158-247 min), the mean aortic crossclamping time was 121.3 min (range, 96-162 min), the mean selective cerebral perfusion time with circulation arrest (including selective cerebral perfusion via right axillary artery) was 64.1 min (range, 48-82 min), and mean left cerebral ischemia time was 11.4 min (range, 8-14 min).

### Postoperative complications and morbidity

Two patients required reexploration sternotomy to check for bleeding due to postoperative coagulopathy and cardiac tamponade. Three patients experienced neurologic deficits, all of whom had acute surgery. Among them, a 67-year-old male with a type A aortic dissection involving the ascending aorta to the bifurcation of the abdominal aorta developed permanent lower limb paraplegia. Postoperative chest computed tomography (CT) revealed a thrombosed false lumen supplying the spinal arteries. The sensation and muscle power of his lower limbs improved with rehabilitation (Table 3).

**Table 3:** Surgical complications

Complication	Number
Reexploration	2

Neurologic deficit	

Transient	2

Permanent	1

Long-term hemodialysis	2

Tracheostomy	2

Transient neurologic deficits consisting of delirium, confusion, and agitation occurred in 2 patients and in both patients resolved before discharge with rehabilitation and medical therapy. Thus, the rate of permanent neurological deficits was 8%.

Hemodialysis was required in 2 patients; both were diagnosed with chronic renal insufficiency before surgery and both required long-term hemodialysis therapy.

Tracheostomy was performed in 2 patients; 1 was diagnosed with vocal palsy after endotracheal extubation, and the other experienced difficulty weaning from the ventilator.

### Postoperative mortality

Two patients died in the postoperative period, resulting in an in-hospital mortality rate of 16.6%. A 64-year-old male with an acute type A aortic dissection extending to the valve received an emergent Bentall procedure and aortic arch replacement. Reexploratory sternotomy was performed on postoperative day 1 due to coagulopathy and the patient died of cardiogenic shock. A 78-year-old male with a type A aortic dissection received emergent aortic arch replacement via a bilateral thoracotomy. Reexploratory sternotomy was performed on postoperative day 1 due to massive bleeding and suspected cardiac tamponade. Postoperatively, the patient could not be weaned from the ventilator and a tracheostomy was performed. The patient died of sepsis and multiple organ failure 2 months after surgery.

## Discussion

In this report, we have presented the results of TAR using a 4-branched graft, DHCA, and SABP in 12 patients. Of the 12 patients, 2 deaths occurred and 1 patient experienced lower extremity paraplegia resulting in an in-hospital mortality rate of 16.6% and a permanent neurological deficit rate of 8%.

Retrograde cerebral perfusion (RCP) with DHCA has been widely used since 1993 [13,14], but sustained neurologic deficits are observed in some patients who receive prolonged DHCA, even with RCP. SABP can be used to deliver oxygenated blood to the brain during DHCA. Sasaki et al. [13] reported a series of 305 patients who received TAR between 2000 and 2005 with SABP via the right axillary artery; 1.6% experienced permanent neurological dysfunction, 6.6% temporary neurological dysfunction, and the mid-term survival rate was 94.6%. The advantages of axillary cannulation include avoiding manipulation of an atherosclerotic femoral artery or calcified ascending aorta and physiological antegrade cerebral perfusion; however, axillary cannulation requires a longer operation time to exposure the whole axillary artery. The local complication rate can be reduced by the use of a side graft for axillary cannulation [15], and local vascular and nerve damage with axillary artery cannulation can be decreased with experience [16].

A criticism of unilateral antegrade cerebral perfusion is inadequacy of perfusion to the contralateral hemisphere; however, transcranial Doppler ultrasound studies have indicated that blood flow with selective cerebral perfusion is satisfactory to maintain the metabolism of both hemispheres [17]. Kucuker et al. [14] reported a total major neurologic event rate of 2.2% in 181 patients who underwent aortic arch replacement with selective cerebral perfusion between 1996 to 2004. Selective cerebral perfusion relies on the patency of the circle of Willis for perfusion of the contralateral hemisphere. In our series, no patients received preoperative studies of the patency of the circle of Willis due to emergent surgical reconstruction and the rate of permanent neurological deficits was 8%. Dossche et al. [18] used unilateral antegrade cerebral perfusion in 37 patients and bihemispheric antegrade perfusion in 69 patients undergoing operations on the proximal thoracic aorta and reported that the type of perfusion was not a predictor of temporary or permanent neurologic dysfunction. Svensson et al. [19] reported that perioperative stroke with focal neurologic deficit was related to the duration of circulatory arrest and to the presence of concurrent distal aortic disease. Because the duration of hypothermic circulatory arrest and increasing age are important predictors of temporary neurologic dysfunction and of fine-motor and memory deficits after aortic arch surgery, reducing the duration of circulatory arrest should reduce the prevalence of these complications [20]. Lastly, while some authorities find it controversial to perform SABP in patients with acute type A dissection due to a higher risk of neurological complications, a number of very recent studies that have shown SABP for acute type A aortic dissection provides acceptable results [21-24].

Our results using a 4-branched graft are comparable to others in the literature. Sakamoto et al. [11] reported the results of TAR using a 4-branched graft in 24 patients with a mean age of 59.4 years. The authors used SABP, continuous cold blood cardioplegia, and open distal anastomosis under circulatory arrest. The hospital mortality rate was 25%, mean total pump time was 204 ± 53 min, aortic crossclamping time was 136 ± 43 min, SABP time was 83 ± 14 min, and circulatory arrest time was 48 ± 10 min. The 5-year actuarial survival rate was 76%. Bednarkiewicz et al. [12] reported use of a 4-branched graft in 6 patients in which the 4^th ^branch of the graft was used for secondary arterial cannulation for continuous brain circulation. Of the 6 patients, 1 death occurred and no patient experienced a permanent neurological deficit. Kulik et al. [25] reported a series of 88 patients who received TAR with DHCA, right axillary artery cannulation and cerebral perfusion, and a presewn multibranched graft and found a 30-day mortality of 5.7%, 3.4% incidence of stroke, 3.4% incidence of spinal cord ischemia, and 3.4% incidence of new-onset renal failure, and a 5-year survival rate of 70.7%.

A 4-branched graft for TAR has several advantages [11,12]. Atherosclerotic lesions near the origin of the arch vessels can be completely resected and graft anastomoses can be performed at the intact distal sites of the arch vessel where dissection has not extended. In Marfan patients, the pathologic portion of the aortic arch can be completely resected. Bleeding from the sites of arch vessel anastomoses can be controlled easily, and the cardiopulmonary bypass time, aortic crossclamping time, and cerebral perfusion time are less as compared to other methods. Also, early establishment of left-side cerebral perfusion from the 4^th ^branch of the graft after the completion of distal arch anastomosis can diminish malperfusion of the left hemisphere. However, there are some disadvantages of using a 4-branched graft. If the arch curve of a 4-branched graft is not well designed, kinking of the bypass graft can occur. In addition, the angle and the size of graft may not be suitable for anastomoses in a particular patient.

Our strategic approach using a 4-branched graft can be summarized as follows. First, establish an arterial cannula at the right axillary or subclavian artery for cardiopulmonary bypass. Second, complete the brachiocephalic trunk and left common carotid artery anastomosis. The anastomosis time for the left common carotid artery is about 10 min; thus the time the whole brain is perfused from only the right subclavian cannula is only approximately 10 min. Third, anastomosis of the distal aortic arch under DHCA and continuous cerebral perfusion from the branched graft. Fourth, anastomosis of the left subclavian artery and ascending aorta replacement with the branched graft.

The primary limitation of this report is the small number of patients and relatively short follow-up time. However, the results are consistent with those of other reports in the literature and confirm the utility of the technique.

## Conclusion

In summary, aortic arch replacement remains a technically challenging procedure. The use of a 4-branched graft, DHCA, and SABP is a useful operative method for aortic arch replacement with acceptable morbidity and mortality.

## Competing interests

The authors declare that they have no competing interests.

## Authors' contributions

All manuscript was written by WLL, and all data of patients were collected by the following surgeons, WLL, CCS, MLL, PL. The collected data were promised by all surgeons. All authors read and approved the final manuscript.
